# The Mediation Role of Moral Personality Between Childhood Psychological Abuse and Cyberbullying Perpetration Attitudes of College Students

**DOI:** 10.3389/fpsyg.2020.01215

**Published:** 2020-06-05

**Authors:** Hang Zhang, Xiaohua Sun, Liang Chen, Hongze Yang, Yandong Wang

**Affiliations:** ^1^School of Humanities and Social Sciences, Beihang University, Beijing, China; ^2^Organization Department of Party Committee, University of Science and Technology Liaoning, Anshan, China; ^3^School of Marxism, University of Science and Technology Liaoning, Anshan, China; ^4^School of Business Administration, University of Science and Technology Liaoning, Anshan, China

**Keywords:** college students, mediating effect, moral personality, childhood psychological abuse, psychological abuse

## Abstract

Guided by social learning theory and general aggression model, this work aims to explore the impact of childhood psychological abuse on cyberbullying perpetration attitudes of college students and the mediating role of moral personality. Using stratified cluster random sampling method, 527 college students were tested by using the Childhood Psychological Abuse Scale, Moral Personality Adjective Evaluation Questionnaire for college students, and the revised Chinese version of Cyberbullying Attitude Questionnaire. Results: (1) Childhood psychological abuse was significantly positively correlated with cyberbullying perpetration attitudes and showed a significant negative correlation with positive moral personality traits. (2) Positive moral personality traits had a significant negative correlation with cyberbullying perpetration attitudes. (3) Positive moral personality traits had a significant mediating effect between childhood psychological abuse and cyberbullying attitudes of college students. The mediating effect accounted for 11.11% of the total effect.

## Introduction

Cyberbullying perpetration is the use of information and communication technology to conduct malicious, repetitive, and hostile behaviors against individuals or groups to harm others (Tokunaga, 2010). Cyberbullying perpetrators always use electronic communication technologies (e.g., social software, mobile short messages, e-mail, and chat rooms) to attack cyberbullying victims. Given the anonymity, time-space, and strong communication of cyberbullying, it seriously affects the physical and mental health of cyberbullying victims (Zych et al., 2018), which leads to emotional disorders, substance abuse, personality disorders, problematic behavior, and even suicide (Lake et al., 2015; Liu et al., 2017, 2019; Yuan et al., 2017; John et al., 2018). Studies found that cyberbullying perpetration among adolescents in secondary school continues when they attend university, and the types of cyberbullying perpetration had a strong consistency (Hinduja and Patchin, 2008). Meanwhile, cyberbullying perpetration has a cross-cultural universality. Beran et al. (2012) surveyed college students in the United States and Canada and found that 33.6% of undergraduates suffered from cyberbullying. According to Jin et al. (2017a), the positive rate of cyberbullying perpetration in Chinese college students was 42.61%. The study of Kokkinos et al. (2014) on 470 Greek college students revealed that cyberbullying perpetration and cyberbullying victims accounted for 58.4% of participants. Further, cyberbullying victims were likely to become new cyberbullying perpetrators, creating a vicious circle (Şerife et al., 2015; Zsila et al., 2019).

While research on cyberbullying perpetration among college students has grown during the last decade (Jenaro et al., 2017), cyberbullying attitudes have remained a relatively unexplored field. Therefore, the limited number of studies on cyberbullying perpetration attitudes is generally exploratory in nature (Wong et al., 2017). Researchers have found that attitudes are linked with behavior (Anderson and Bushman, 2002; Boulton et al., 2012; Barlett et al., 2016). Barlett et al. (2016) and Barlett (2017) suggested that more accepting attitudes toward the anonymity and strength imbalance belief predicted more positive cyberbullying perpetration attitudes, which in turn predicted cyberbullying perpetration. Heirman and Walrave (2011) found that cyberbullying attitudes were the strongest predictor of cyberbullying intentions. Higher cyberbullying intentions predicted more frequent cyberbullying perpetration. Therefore, cyberbullying perpetration attitudes could be a stronger predictor of cyberbullying perpetration than other individual factors. Given that cyberbullying intervention programs would do well to promote less positive attitudes toward cyberbullying perpetration and perpetrators (Boulton et al., 2012), the aim of this study was to explore cyberbullying perpetration attitudes by investigating their mechanisms.

### Childhood Psychological Abuse and Cyberbullying Perpetration Attitudes

In previous studies, researchers emphasized the impact of family factors on cyberbullying (Charalampous et al., 2018; Yang et al., 2018). The social learning theory (SLT) states that parents play an important role in shaping children’s aggressive behavior (Bandura, 1978). Studies have shown that childhood psychological abuse was an important predictor of cyberbullying perpetration among college students (Jin et al., 2017a). Childhood psychological abuse refers to the continuous and repeated adoption of a series of inappropriate behaviors by adults, including the five types of behaviors, namely, terrorizing, ignoring, belittling, intermeddling, and corrupting, which causes tremendous damage to children’s cognitive, emotional, and behavioral development (Pan et al., 2010). Studies have reported that approximately 45.3% of Chinese college students suffered psychological abuse during childhood (Xie et al., 2008). These college students had significantly higher levels of loneliness and depression than ordinary college students (Song and Liu, 2013; Gardner et al., 2014; Baugerud et al., 2016), and were accompanied by social emotional development obstacles, poor peer relationships, and social difficulties (Cecil et al., 2014; Liu et al., 2016, 2017). Although previous studies have proven that a significant positive correlation exists between childhood psychological abuse and cyberbullying perpetration among college students (Jin et al., 2017a), to our knowledge, no studies have examined the relationship between childhood psychological abuse and cyberbullying perpetration attitudes. Given that cyberbullying perpetration attitudes had a significant positive predictive effect on the occurrence and frequency of cyberbullying perpetration (Fanti et al., 2012; Barlett, 2017), we hypothesized that childhood psychological abuse positively predicts the cyberbullying perpetration attitudes of college students (Hypothesis 1).

### Moral Personality as a Mediator

The general aggression model (GAM) focuses on the individual and situational factors that influence aggressive behavior (Anderson and Bushman, 2002). Individual factors include personality traits, motives, attitudes, beliefs, values, behavioral scripts, long-term goals, and any other consistent characteristics the individual brings to the situation (Kowalski et al., 2012). In accordance with the GAM, moral personality was taken into consideration as potential protective factors in the association between childhood psychological abuse and cyberbullying perpetration attitudes (Anderson and Bushman, 2002; Kowalski et al., 2012). In the past few years, researchers have paid increasing attention to the influence of personality on cyberbullying behavior. As the moral dimension of personality, moral personality is the overall organization of moral cognition, emotion, and behavior formed by individuals in the process of socialization and the unity of an individual’s inner quality and external moral behavioral mode (Wang, 2009). Research showed that the positive moral personality of college students were significantly negatively correlated with anti-social behaviors (e.g., violation, addiction, and attack tendencies; Zhang, 2012). In the developmental period of moral personality, as a childhood experience, childhood psychological abuse was closely related to individual personality characteristics (Gabalda et al., 2009; Martinotti et al., 2009; Conger et al., 2010; Auslander et al., 2016). Studies have shown that psychological abuse was negatively related to extroversion, and individuals who experienced more psychological abuse showed high levels of neuroticism, impulsion, and other negative personality tendencies (Li et al., 2017). Jin and Wang (2017) revealed that personality played a mediating role in childhood psychological abuse and aggression. Therefore, it could be posited that positive moral personality played a mediating role in the relationship between childhood psychological abuse and the cyberbullying perpetration attitudes of undergraduates (Hypothesis 2).

### The Present Study

In summary, although previous studies have found a positive relationship between childhood psychological abuse and cyberbullying perpetration, few have discussed its mechanisms and examined the roles of family and individual psychological factors in the mechanisms of the cyberbullying perpetration attitudes of college students. Positive psychologists emphasized that protective factors, such as moral personality, had a positive impact on mental health, well-being, and life satisfaction (Tweed et al., 2011; Kwok et al., 2016). On the basis of the intervention for cyberbullying perpetration among undergraduates, the mediation effect of positive moral personality should be examined in order to provide theoretical support and empirical evidence.

## Materials and Methods

### Participants

The stratified cluster random sampling method was used to select 620 freshmen to seniors from a university in Liaoning province as the research objects. We used self-study time to carry out the test in classrooms and collected the questionnaires on the spot.

All participants were ordinary college students. After excluding invalid questionnaires with inclusion and exclusion criteria (25 questionnaires with the same responses to all questions, and 23 questionnaires missing more than half of the data), 572 valid questionnaires were obtained, and the effective recovery rate was 92.25%. There were 347 males (60.7%) and 252 females (39.3%), and 263 with urban residence registration (46%) and 309 with rural household registration (54%). The ages of participants ranged from 17 to 23 years of age (*M* = 20.49, SD = 1.44). The study protocol was approved by the Research Ethics Committee of the University of Science and Technology Liaoning (China).

### Measures

#### Children’s Psychological Abuse Scale

The Children’s Psychological Abuse Scale compiled by Pan et al. (2010) was used. The scale has 23 items, using a five-point Likert scale, where 0 stands for “No,” and 4 stands for “Always.” The scale includes five subscales, namely, threatening (e.g., My parents were angry with me), ignoring (e.g., My parents didn’t answer my questions), belittling (e.g., My parents scolded me for no reason), intermeddling (e.g., My parents looked in my diary), and indulging (e.g., My parents didn’t forbid me to drink). In this study, the Cronbach α coefficient of the scale was 0.90, and the five subscales of Cronbach α coefficients were 0.54–0.87.

#### Moral Personality Adjective Evaluation Questionnaire for College Students

The Moral Personality Adjective Evaluation Questionnaire for College Students (MPAEQ; Wang, 2009) consists of 72 items. In this study, we used the subscales of positive moral personality traits (35 items). The subjects should be assessed on a scale of 1–5 according to their own level, where 1 means “Not so consistent” and 5 means “Very consistent.” Three factors can be measured, namely, kindness, selflessness, and honesty-thriftiness. Among them, kindness includes three subscales, namely, benevolence, faith, and respect; selflessness consists of two subscales, namely, integrity and selflessness; and honesty-thriftiness consists of two subscales, namely, honesty and diligence-frugality. The Cronbach α coefficients of the three dimensions were 0.79–0.94.

#### The Chinese Revised Version of the Cyberbullying Attitude Measure

We used the Cyberbullying Attitude Measure (CAM) compiled by Barlett et al. (2016). We initially obtained Barlett’s revision authorization for the scale and then revised the Chinese version of the CAM on the basis of the standards of the International Test Commission. The Chinese version of CAM contains 10 items using a five-point Likert scale, where 1 means “Completely disagree,” and 5 means “Completely agree.” A higher score implies a stronger individual’s positive attitude toward cyberbullying perpetration. The questionnaire includes two subscales, namely, harmful cyberbullying attitudes (e.g., It is alright to send harmful online messages/posts to another) and general cyberbullying attitudes (e.g., Attacking others online can be justifiable), each containing five items. In this study, the Cronbach α coefficients of the harmful and general cyberbullying attitude subscales were 0.75 and 0.87, respectively.

### Data Analysis

SPSS version 23.0 was used for descriptive statistics, reliability analysis, and correlation analysis between variables. Mplus version 8.1 was used to analyze the structural equation modeling. The missing values were replaced with the series mean method in SPSS. The series mean refers to replacing missing values with the mean for the entire series.

## Results

### Correlation Analysis Between Variables

The descriptive statistics and related analysis of childhood psychological abuse, positive moral personality traits, and cyberbullying perpetration attitudes were conducted. The results of the correlation analysis showed that a significant correlation existed among childhood psychological abuse, positive moral personality traits, and cyberbullying perpetration attitudes (see Table 1). Particularly, childhood psychological abuse was significantly positively correlated with cyberbullying perpetration attitudes, and negatively correlated with positive moral personality traits. Moreover, a significant negative correlation existed between positive moral personality traits and cyberbullying perpetration attitudes.

**TABLE 1:** Correlation matrices in key variables (*N* = 572).

Variables	*M*	SD	1	2	3
1 Childhood psychological abuse	0.83	0.54	1		
2 Positive moral personality traits	3.78	0.52	−0.18**	1	
3 Cyberbullying perpetration attitudes	1.86	0.66	0.40**	−0.25**	1

**p < 0.01.

### Mediation Analysis

**FIGURE 1:**
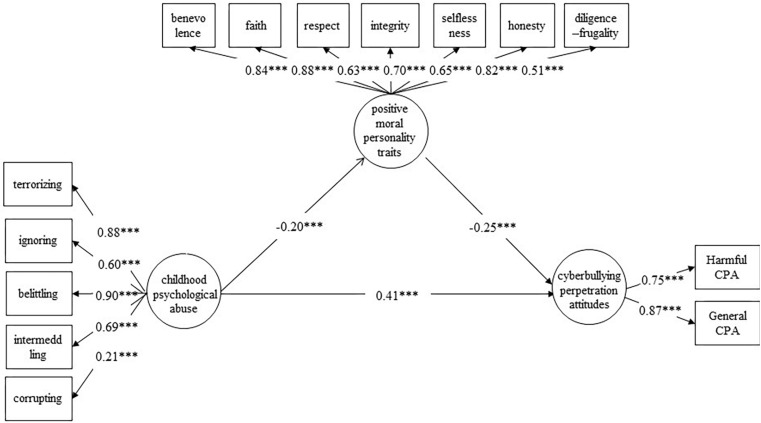
Positive moral personality traits as a mediating variable between childhood psychological abuse and cyberbullying perpetration attitudes.

In order to test the hypothesized mediation model (see Figure 1), we used structural equation modeling. To evaluate model fit, we used several frequently used goodness-of-fit indices: the chi-squared goodness-of-fit statistic (χ*^2^*), the comparative fit index (CFI), the Tucker–Lewis Index (TLI), the standardized root mean square residual (SRMR), and the root mean square error of approximation (RMSEA). A value of 0.90 or higher for CFI and TLI implies an acceptable fit; SRMR and RMSEA values of less than 0.08 indicate a moderate fit (MacCallum et al., 1996).

A bootstrapping and Markov chain Monte Carlo (MCMC) method was used in this study to test the multiple mediating effects of positive moral personality traits. First, the significance of the total effect (Model A) was examined. In this study, the total effect of childhood psychological abuse on the cyberbullying perpetration attitudes of college students was 0.45, and the total effect coefficient was significant (*p* < 0.001). As expected, the fit indices of Model A are presented in Table 2. Although RMSEA was not within the acceptable cutoff (0.08), TLI, CFA, and SRMR were acceptable to support our hypothesized mediation model.

**TABLE 2:** The fitting index of the models.

Model	χ*^2^*	*df*	CFI	TLI	SRMR	RMSEA
Model A	78.043***	13	0.96	0.93	0.05	0.09
Model B	256.549***	74	0.95	0.94	0.05	0.07

***p < 0.001.

Second, the significance of the path coefficients was checked. Model B was initially constructed (see Figure 1); childhood psychological abuse was taken as an independent variable, cyberbullying perpetration attitudes as a dependent variable, and positive moral personality traits as a mediator variable. The analysis showed that all path coefficients reached significant levels (*p* < 0.001), and the normalized factor loadings of the observed variables reached a significant level (*p* < 0.001). The fitting indices of Model B were acceptable (see Table 2). Therefore, positive moral personality partly mediated the influence of childhood psychological abuse on the cyberbullying perpetration attitudes of college students.

Finally, the confidence interval of the path coefficients was estimated, and we used 1000 bootstrap sampling iterations with replacement. The results showed that the bootstrap 95% confidence interval (0.02, 0.086) for the total indirect effect of positive moral personality traits did not contain 0. This finding indicated that positive moral personality traits had a significant mediating effect between the childhood psychological abuse and cyberbullying attitudes of college students. The mediating effect accounted for 11.11% of the total effect. The results supported our hypothesized multiple mediation model.

## Discussion

### Direct Effect of Childhood Psychological Abuse on Cyberbullying Perpetration Attitudes

The results of this study indicated that childhood psychological abuse has a significantly positive effect on the cyberbullying perpetration attitudes of college students, supporting Hypothesis 1. Our finding was consistent with those of previous studies. Jin et al. reported that childhood psychological abuse was significantly positively correlated with cyberbullying among college students (Jin et al., 2017a, b). The GAM posits that the generation of cyberbullying is the result of the interaction between the individual and the environment (Anderson and Bushman, 2002; Kowalski et al., 2012). While the individual was affected by the environmental context, the environmental factors are internalized or processed, and the external stimulus is evaluated. When an external stimulus is threatening or the evaluation is unsatisfactory, the individual will generate aggressive behavior (Zurbriggen et al., 2010; Jin and Wang, 2017; Zhang et al., 2017). The SLT states that parents are important role models for children’s learning (Bandura, 1978). In abusive families, parents’ attacks on children, such as terrorizing, ignoring, and belittling, make children think that attack is a suitable communication approach and they may learn through imitation. The communication style of individuals is constantly strengthened by their parents, and individuals will show aggressive behavior in interpersonal communication. Given the convenience and anonymity of the network, college students with childhood psychological abuse are more likely to use network, mobile communication devices, and other tools to resolve interpersonal conflicts or violations, thereby exhibiting a high level of cyberbullying perpetration attitudes. Therefore, individuals who are psychologically abused during childhood are more likely to develop cyberbullying perpetration attitudes in adulthood.

### Mediating Role of Positive Moral Personality Traits

The results of this study showed that childhood psychological abuse had an indirect effect on the cyberbullying perpetration attitudes of college students through positive moral personality, with an effect rate of 11.11%, supporting Hypothesis 2. The results showed that childhood psychological abuse experiences were more likely to hinder the development of positive moral personality traits, which are the protective factors to the formation of cyberbullying perpetration attitudes under psychological abuse. Research concerning parental styles has indicated that children with negative parenting are likely to participate in cyberbullying, as they are exposed to cyberspace without supervision or as a way to vent bad emotions (Charalampous et al., 2018). For example, an authoritarian parenting style is also closely related to cyberbullying. According to SLT (Bandura, 1978), parent-child interactions in the family environment are of vital importance for the development of children, who learn to bully disadvantaged peers by watching these interactions between their family members (Charalampous et al., 2018).

The results of this study were also consistent with previous studies showing that moral identity as a personal factor had an direct influence on adolescents’ aggression and bullying (Low and Espelage, 2013), and was negatively related to cyberbullying perpetration behavior among Chinese adolescents (Yang et al., 2018). The SLT (Bandura, 1978) states that children who suffer long-term mental damage in abusive environments, such as terrorizing, belittling, and ignoring, can be hindered in their normal psychological development and are often hostile or anxious during social interactions. Over time, positive moral personality traits can become difficult to form, which can increase the possibility of attack behavior (Liu et al., 2012). In addition, a good parent-child relationship is an “internal working mechanism” for children to adapt to society and handle various interpersonal relationships in the future. The social development of college students becomes more sluggish than their peers because this “internal working mechanism” is destroyed; they feel inferior, sensitive, and become suspicious, which leads to the weakening of the self-regulatory function of moral adjustment and means that they can hardly form positive moral personalities. On the contrary, individuals with high levels of positive moral personality often make well-meaning explanations of other people’s behavior or speech, and adopt adaptive emotion regulation strategies to show fewer aggressive behaviors (Jin et al., 2017a, b).

### Implications

The model of this study would allow us to gain a better understanding of the pathways through which parenting affects students’ acceptance in cyberbullying perpetration, and would contribute to the development of intervention programs designed to prevent cyberbullying perpetration. Based on the results, anti-cyberbullying interventions in schools should call for a stronger role for moral personality education catering to the individual needs of the students in implementing a cyberbullying prevention plan. This would make these staff members not only attach importance to professional training, but also strengthen the cultivation of students’ moral personality traits. Another implication for parents is that avoiding adverse early rearing environments is likely to reduce the severity of positive cyberbullying perpetration attitudes. It will be essential for parents to adopt scientific content and enrich the content of family education to improve parent–child relationships (Doty et al., 2018).

### Limitations

Several limitations of the present study should be noted. The first limitation is that a cross-sectional study precluded causal interpretations. It will be important for future investigations to replicate our findings using longitudinal research to collect data at several time points. The second limitation is the use of self-reporting methodology. Using only respondents as the source enhances the risk of reliability through error-inflating method variance (Kajonius and Björkman, 2020). Future studies may attempt to use an interview method to test the effects of individual and situational factors in the mechanisms of cyberbullying perpetration attitudes. The third limitation is that the Cyberbullying Attitude Measure did not measure behavioral factors such as cyberbullying perpetration. Although previous studies have confirmed that attitudes and behavior were linked (Anderson and Bushman, 2002; Barlett et al., 2016; Barlett, 2017), future research should attempt to establish a model to investigate the effect of influential factors on cyberbullying perpetration.

## Conclusion

In summary, the present study investigated a mediating model to examine the relationship between childhood psychological abuse and cyberbullying perpetration attitudes. The findings suggested that childhood psychological abuse was positively associated with cyberbullying perpetration attitudes. Moreover, positive moral personality played a mediating role in the relationship between childhood psychological abuse and cyberbullying perpetration attitudes among Chinese college students.

## Data Availability Statement

The datasets generated for this study are available on request to the corresponding author.

## Ethics Statement

The studies involving human participants were reviewed and approved by the Research Ethics Committee of the University of Science and Technology Liaoning (China). Written informed consent to participate in this study was provided by the participants.

## Author Contributions

HZ, XS, and LC performed the experiments. YW analyzed the data. XS and LC contributed reagents, materials, and analysis tools. HZ, LC, and HY wrote the manuscript.

## Conflict of Interest

The authors declare that the research was conducted in the absence of any commercial or financial relationships that could be construed as a potential conflict of interest.
